# The Carbon Emission Assessment of a Building with Different Prefabrication Rates in the Construction Stage

**DOI:** 10.3390/ijerph19042366

**Published:** 2022-02-18

**Authors:** Qingye Han, Junjie Chang, Guiwen Liu, Heng Zhang

**Affiliations:** School of Management Science and Real Estate, Chongqing University, Chongqing 400044, China; hanqy11@cqu.edu.cn (Q.H.); 20150499@cqu.edu.cn (J.C.); bournezhang@cqu.edu.cn (H.Z.)

**Keywords:** carbon emission, prefabrication rate, construction industry, construction stage, coefficient method

## Abstract

The utilization of prefabricated components is taken as a potential way to reduce carbon emissions from the construction industry, and the prefabrication rate may be a factor that influences the mitigation efficiency. This study develops an assessment method to compare carbon emissions of a building in the construction stage when it is built with multiple different prefabrication rates. Firstly, two carbon sources (building materials and machineries) and three construction sub-phases (production of materials and components, transportation, and on-site construction) are determined to clarify the calculation boundary. Then, a carbon emission measurement model for prefabricated buildings in the construction stage is developed by using a process-based method. A dormitory building in Chongqing, China, is selected to conduct a case study to show the application of the provided model. The result shows that the carbon emission of prefabricated buildings is higher compared to that of traditional cast-in situ buildings. Moreover, the emission of prefabricated buildings decreased slightly with the increase in the prefabrication rate. A detailed discussion is followed to investigate the reason why the carbon emission does not decrease with the utilization of prefabricated units. Based on the discussion, some suggestions are given to improve the carbon emission reduction efficiency of prefabrication techniques.

## 1. Introduction

It is a necessary revolution for human beings to accelerate the green and sustainable development. Climate change caused by greenhouse gas is one of the most urgent environmental problems faced by mankind. According to the United Nations Intergovernmental Panel on climate change (IPCC), the temperature increase caused by human activities is about 1.0 °C compared to the pre-industrial era. If the current rise rate continues, the mean temperature will increase by 1.5 °C between 2030 and 2052 [1]. Carbon emissions from the building industry are one of the most important sources of greenhouse gases [2]. According to statistics, energy consumption and CO_2_ emission from building construction and operation accounts for 35% of the global energy consumption and 29% of the global total CO_2_ emissions [3]. China is in the midst of massive construction, and carbon emissions from the construction industry even take up about 50% of the national carbon emissions. Relevant studies show that the carbon emission peak of China’s construction industry will appear in 2035, which is significantly delayed compared to the national target of carbon peak by 2030 [4]. How to reduce carbon emissions from the construction industry becomes more and more important in China.

Existing studies on the building carbon emission can be generally divided into macro and micro aspects. Most macro-level research [5,6,7] pays more attention to the impact of clean energies and new materials on the carbon emissions of the whole industry. For example, Du et al. [6] conducted comparative research on the carbon emissions of the building industry in multiple provinces of China and concluded that promoting low-carbon building technology and reducing high-carbon materials could reduce carbon emissions from the industry; Chen and Chen [5] studied the potential impact of coal substitution policy on carbon emission of the building industry in the 13th Five Year Plan period of China and concluded that the carbon emission would decline by 20 to 29%. The micro-level research [8,9,10,11] focuses on how to reduce carbon emissions of some specific buildings by changing materials, structures, construction mode, etc. For example, Geng et al. [10] found that the carbon emissions of a building can be reduced by 0.16–2.85 t per unit using wood flooring instead of ceramic tiles; Chen et al. [8] found that the CO_2_ emissions of a case building can be reduced 86t per year by improving Thermal envelope. Note that this paper concerns the micro-level building carbon emission.

From the view of the life cycle of a building, the carbon emissions in the operation stage account for more than 60% [12,13,14,15]. Therefore, the existing research on building carbon emissions mainly focus on the whole life cycle [16,17,18,19,20,21] or only the operation stage [22,23,24,25,26]. Due to a large number of energy saving laws, regulations and other measures, the carbon emission in the operation stage of a building is declining sharply, and the proportion of carbon emission in the construction stage is gradually increasing [27,28]. Although carbon emissions in the construction stage are relatively lower than that in the operation stage, it cannot be ignored because the energy consumption and carbon emission are more concentrated in this shorter period than that in the operation stage [29]. In the construction stage, the proportion of traditional cast-in situ subdivisional works is decreasing, while that of prefabricated subdivisional works is increasing [30]. Compared with the traditional cast-in situ construction method, prefabricated buildings are considered to be more cost effective and energy saving [28,31]. Moreover, it can reduce construction waste [32] and environmental damage [33]. Both the central and local governments have made great efforts to replace cast-in situ with prefabrication mode in China [34]. Unlike in other developed countries, the promotion of prefabricated buildings is still in its infant stage in China [35]. Whether the prefabricated technique can help reduce carbon emissions needs intensive research.

In the construction stage, some comparative studies on carbon emissions of traditional cast-in-place buildings and prefabricated buildings have been conducted. Mao et al. [36] selected a prefabricated building and a cast-in situ building constructed by different contractors in Shenzhen as case studies and finally concluded that prefabrication construction can reduce the building carbon emissions by about 30 kg/m^2^. Dong et al. [37] took two similar apartments in Hong Kong for comparative case studies, and concluded that prefabricated facades could reduce carbon emissions by 2.1 kg/m^2^. Ji et al. [38] compared the carbon emissions of two buildings in Chongqing, and drew the conclusion that the on-site prefabrication mode could reduce carbon emissions by 3.1%. Sandanayake et al. [39] selected a cast-in-place office building and a prefabricated office hotel in Chengdu for comparison, and concluded that the prefabricated mode can reduce carbon emission by 8.40% compared with the cast-in-place mode. Du et al. [40] selected two residential buildings with similar structures in Xi’an to conduct comparative case study and came to a conclusion that the prefabricated building could reduce carbon emissions by 18%.

In a summary, existing literature about the comparison of carbon emissions from cast-in situ buildings and prefabricated buildings in the construction stage in the specific background of China are few. In addition, most studies take two or more similar buildings as cases for comparison, while different design, data collection methods, etc., may reduce their comparability. Furthermore, prefabricated buildings do not mean that everything is precast, and the prefabrication rate will influence the carbon emission. At present, there is a lack of comparative research on carbon emissions with different prefabrication rates of a specific building in the construction stage. The contributions of this paper are as follows:

(1) The carbon emission measurement boundary in the construction stage of prefabricated buildings is determined.
(2) A method to measure carbon emissions of buildings with different prefabrication rates in the construction stage is designed.
(3) Unlike two or more case buildings in other research, the same building case is selected to show the effectiveness of the model-based method.
(4) Based on the case results, suggestions for carbon emission reduction of the construction industry are put forward in the specific context of China.

The remainder of this paper is organized as follows. The research methodology is introduced in Section 2, followed by a dormitory building case study to show the application and effectiveness of the presented method in Section 3. The result analysis is conducted in Section 4. Discussions including comparison with other studies, proposed method, and corresponding suggestions are conducted in Section 5. The conclusion of the whole paper is summarized in Section 6.

## 2. Methodology

### 2.1. Definition of the Assessment Boundary

#### 2.1.1. Stage Boundary of the Assessment

**Figure 1:**
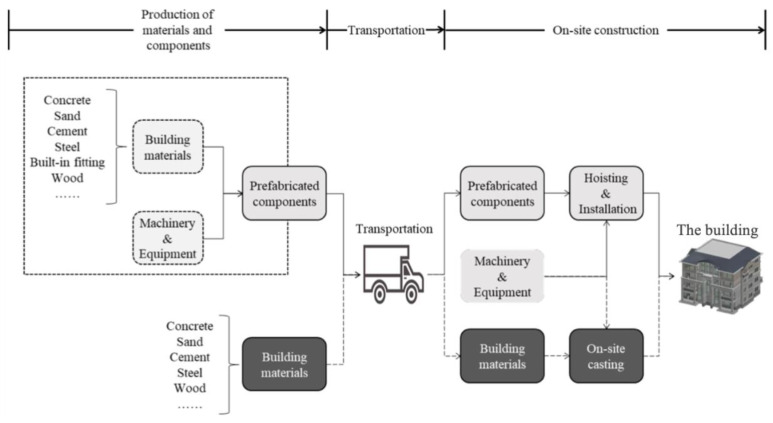
Three Phases of the Construction Stage.

The entire life cycle of buildings includes multiple stages, such as the design, the construction, the operation, and the deconstruction. Note that this study focuses on the construction stage of prefabricated buildings. As shown in Figure 1, the whole construction stage can be divided into three phases: (1) the production of materials and components, (2) transportation, and (3) the on-site construction. This process division lays the foundation for carbon source identification.

#### 2.1.2. Space Boundary of the Assessment

**Figure 2:**
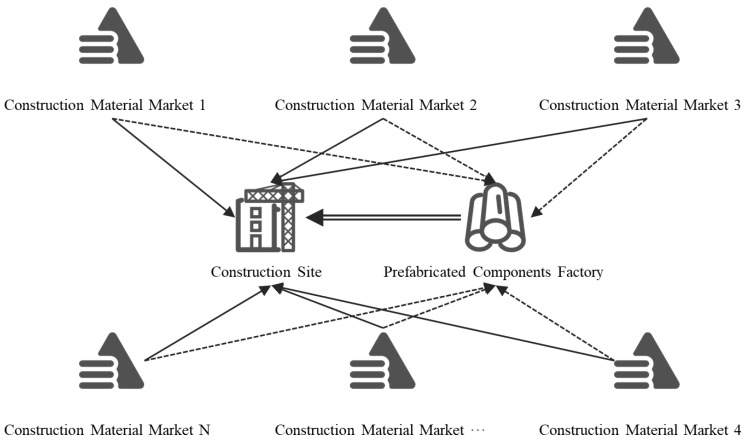
The Space Boundary of the Assessment.

The assessment space in this paper involves all locations where construction related activities happen for completing a project. More precisely, it includes the production plant of materials and prefabricated components, the transportation road, and the construction site of the project. As shown in the Figure 2, it is a network of spaces that exist for a specific project.

#### 2.1.3. The Boundary of Carbon Emission

Carbon emission is related to climate change. Many researchers use the concept of greenhouse gases when studying climate change. It includes Carbon Dioxide (CO_2_), Methane (CH_4_), Nitrous Oxide (N_2_O), Chlorofluorocarbons (CFCs), Ozone (O_3_), Hydrofluorocarbons (HFCs), Per fluorocarbons (PFCs), and Sulfur Hexafluoride (SF_6_). Among these harmful gases, CO_2_ is the main greenhouse gas, and the carbon emission in this paper is limited to the emission of CO_2_.

### 2.2. Selection of the Assessment Method

In previous studies, there are four methods that are mainly employed to assess the environmental impact of buildings. The first one is the statistical analysis which is based on sufficiently comprehensive published statistics. However, it is difficult to collect detailed data in most countries, and in turn this method is not available in most studies. The second one is the input–output analysis, which is a top-down method on a macroeconomic scale. The third one is the process-based analysis, which is a bottom-up method based on the production process of goods or services. The fourth hybrid analysis combines the above mentioned methods according to specific research objectives. Although the prefabrication is undergoing a rapid development, its complete input–output data are still unavailable in China. The more feasible approach is to employ the micro method to calculate carbon emissions. Therefore, a process-based analysis is selected for this study. For process-based analysis, the following three carbon emission assessment methods are commonly adopted in the construction industry. Their features are summarized in Table 1:

(1) Coefficient method. It equals the product of carbon source activities and their corresponding carbon emission factors for a specific building;
(2) Mass balance method. The carbon difference between input and output of all materials used in the production process;
(3) Actual assessment method. The carbon emission data of a building in the construction stage is collected on site.

Considering the summarized features in Table 1, the carbon emission assessment model will be developed by using the coefficient method, which is proposed by the Intergovernmental Panel on Climate Change (IPCC). According to the coefficient method, the carbon emission can be expressed as follows:

(1) $$E=\sum_{i=1}^{n}a_{i}f_{i},$$

where *E* refers to the total carbon emission of a prefabricated building in its construction stage from *n* kinds of carbon emission activities, $a_{i}$ indicates the amount of the *i*th carbon emission activity, and $f_{i}$ represents the factor of the *i*th carbon emission activity per unit.

### 2.3. Carbon Sources and Emission Factors

#### 2.3.1. The Identification of Carbon Sources

The identification of carbon sources is based on specific industry characteristics and manufacturing process. Based on existing achievements, the sources of carbon emission in the construction stage of prefabricated buildings are classified into the following three aspects:

(1) The carbon emission due to materials’ consumption from machineries. It generally includes steel, concrete, and other materials. This part of carbon emission comes from the production process of raw materials and can be calculated by multiplying the amount of materials by the corresponding carbon emission factors.
(2) The carbon emission caused by the energy consumption. For example, fuel, electricity, water, and other energies. This kind of carbon emission can also be calculated by multiplying the amount of energy consumption by its corresponding carbon emission factor.
(3) The carbon emission from man-power. It equals to the number of workers and the number of hours and the amount of carbon emissions they breathe per unit time.

According to professional engineers, it is found that carbon emission from man-power breathing of different construction methods is very limited, so the carbon emissions from man-power will not be included during the calculation process, and only carbon emissions from materials and machinery are defined in this paper.

Considering the three phases in Figure 1 and the above-mentioned carbon sources, the specific carbon emission sources ($E_{i-j}$) of prefabricated buildings in the construction stage are defined as follows. Herein, i∈{1,2,3} and j∈{1,2} indicate the three phases and the two kinds of carbon sources, respectively. It should be noted that $E_{1-1}=E_{1-1_{a}}+E_{1-1_{b}}$:

- $E_{1-1}$: Carbon emissions from materials in the first phase;
- $E_{1-1_{a}}$: Carbon emissions from materials used for component production;
- $E_{1-1_{b}}$: Carbon emissions from materials used for cast-in-place construction;
- $E_{1-2}$: Carbon emissions from machineries for the component production in the first phase;
- $E_{2-1}$: Carbon emissions from materials used in the transportation phase;
- $E_{2-2}$: Carbon emissions from machineries used in the transportation phase;
- $E_{3-1}$: Carbon emissions from materials used in the on-site construction phase;
- $E_{3-2}$: Carbon emissions from machineries in the on-site construction phase.

#### 2.3.2. The Determination of Carbon Emission Factors

By summarizing existing authoritative and high-cited literature [36,41,42], the carbon emission factors used in this paper are shown in Table 2.

### 2.4. The Assessment Model of Carbon Emissions of a Prefabricated Building

The assessment model of carbon emissions of a prefabricated building in the construction stage is composed of the following formulas:

(1) The total carbon emission in the construction stage:

(2) $$E=\sum_{i=1}^{3}\sum_{j=1}^{2}E_{i-j}=\sum_{i=1}^{3}E_{i}=\sum_{j=1}^{2}E_{j}^{{}^{\prime }},$$

where:

- *E*: The total carbon emission (unit: kg);
- $E_{i}$: The total carbon emission in the *i*th phase of the construction stage (unit: kg);
- $E_{j}^{{}^{\prime }}$: The total carbon emission of the *j*th carbon source in the construction stage (unit: kg).

(2) The carbon emission from materials in the *i*th phase of the construction stage:

(3) $$E_{i-1}=\sum_{p=1}^{m}\sum_{q=1}^{n}\left[\left(\frac{A_{p}}{A_{p}^{{}^{\prime }}}\times M_{pq}\right)\times f_{q}\right],$$

where:

- p∈{1,2,3,⋯,m} means that the construction project can be divided into *p* subdivisional works;
- q∈{1,2,3,⋯,n} means that the construction project needs *q* kinds of materials;
- $A_{p}$: The real engineering quantity of the *p*th subdivisional work;
- $A_{p}^{{}^{\prime }}$: The quota engineering quantity of the *p*th subdivisional work;
- $M_{pq}$: The consumption of the *q*th material per quota engineering quantity in the *p*th subdivisional work;
- $f_{q}$: The carbon emission factor of the *q*th material.

(3) The carbon emission from machineries in the *i*th phase of the construction stage:

(4) $$E_{i-2}=\sum_{p^{=}1}^{m}\sum_{q^{{}^{\prime }}=1}^{n^{{}^{\prime }}}\left[\left(\frac{A_{p}}{A_{p}^{{}^{\prime }}}\times M_{pq^{{}^{\prime }}}\times C_{q^{{}^{\prime }}}\right)\times f_{q’}\right],$$

where:

- $q^{{}^{\prime }}\in \left\{1,2,3,\cdots ,n^{{}^{\prime }}\right\}$ means that the construction project needs $q^{{}^{\prime }}$ kinds of machineries;
- $M_{pq^{{}^{\prime }}}$: The consumption of the $q_{\mathrm{th}}^{{}^{\prime }}$ machinery per quota engineering quantity in the *p*th subdivisional work;
- $C_{q}^{{}^{\prime }}$: The energy consumption per $M_{pq^{{}^{\prime }}}$;
- $f_{q{}^{\prime }}$: The carbon emission factor of the $q_{\mathrm{th}}^{{}^{\prime }}$ energy;
- The meanings of $A_{p}$, $A_{p}^{{}^{\prime }}$, *p*, and *m* are as the same as that in the above equation.

(4) The total carbon emission in the *i*th phase of the construction stage:

(5) $$E_{i}=\sum_{j=1}^{2}E_{i-j}=E_{i-1}+E_{i-2},$$

where $E_{i-1}$ and $E_{i-2}$ can be calculated from Equation (3) and (4), respectively.

(5) The total carbon emission of the *j*th sources in the construction stage:

(6) $$E_{j^{{}^{\prime }}}=\sum_{i=1}^{3}E_{i-j}=E_{1-j}+E_{2-j}+E_{3-j},$$

where $E_{1-j}$, $E_{2-j}$, and $E_{3-j}$ can also be calculated from Equations (3) and (4).

## 3. Case Study

**Figure 3:**
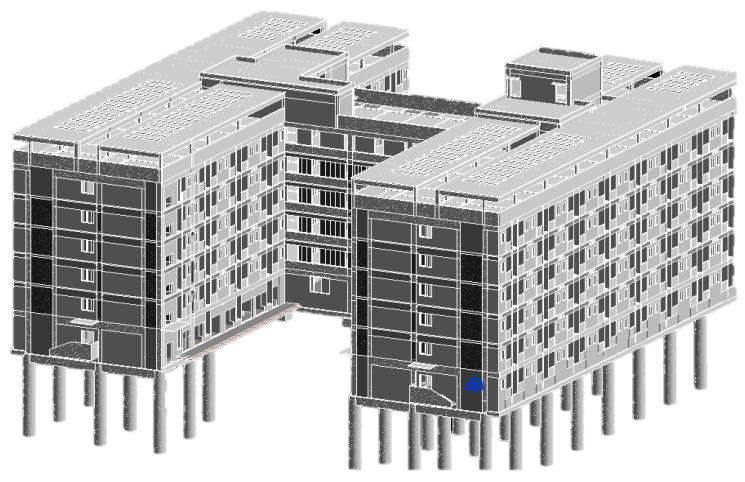
The BIM Model of the Case Building.

In this paper, a dormitory building project of Chongqing Jianzhu College from Chongqing, China is taken as an example to show how the prefabrication rate influence the carbon emission. The Building Information Modeling (BIM) technique is used to carry out potential component replacement and corresponding engineering quantity statistics. The features of the building are shown in Table 3, and its BIM model of the building is shown in Figure 3.

According to the design of the building, the cast-in-place part includes the underground, first, and top floor, and prefabricated components are utilized in the left middle floors. When different prefabricated components are used, the prefabrication rate will also be different. By combining different prefabricated components, four rates results are obtained and shown in Table 4. Based on an official industrial standard in China, the “Evaluation Standard for Prefabricated Buildings (GB/T51129-2015)”, the prefabrication rate is calculated according to the following formula:

(7) $$R=\frac{V_{1}}{V_{2}}\times 100\%,$$

where *R* refers to the prefabrication rate of the building, $V_{1}$ (unit: m^3^) represents the concrete volume used by prefabricated components, $V_{2}$ (unit: m^3^) indicates the total concrete volume utilized by both prefabricated components and cast-in-place activity on ground.

As shown in Table 4, the traditional cast-in-place building has no prefabricated components, and thus the prefabrication rate is 0; when stairs, beams, and slabs are prefabricated, the prefabrication rate is 22.86%; when stairs, beams, slabs, columns, and part of the shear walls are prefabricated, the prefabrication rate is 32.69%; if stairs, beams, slabs, columns, some shear walls, and partition boards are prefabricated, the prefabrication rate is 46.98%.

Based on the engineering drawings and the BIM, the engineering quantities of the case building with different prefabrication rates can be obtained. According to “The Engineering Valuation Quota of Housing Construction and Decoration in Chongqing (CQJZZSDE-2018)”, the materials’ and machineries’ consumption data can be obtained. The energy consumption data can be collected from “The Machinery Quota of Construction Engineering in Chongqing (CQJXDE-2018)”. Combining the above three steps, the carbon emission factors in Table 2, and the model in Section 2, the carbon emissions of the case building with different scenarios are obtained and shown in Table 5. To make it clear, the unit of carbon emission here is kg/m^2^.

Herein, the prefabrication rate of 22.86% (the third column in Table 5) is taken as an example to show the calculation process. In this background, some stairs, beams and slabs are prefabricated components, while other left subdivisional works are cast-in-place. To make it simplified, only the carbon emission of prefabrication part is calculated here because the assessment processes of the two parts are similar. Based on the BIM and engineering drawing of the case, four kinds of real engineering quantities ($A_{p}$) of prefabrication subdivisional works in three phases are shown in Table 6.

The carbon emission calculation processes of stairs, beams, and slabs are also similar, only the assessment process of prefabricated stairs is shown for simplification. The quota engineering quantities ($A_{p}^{{}^{\prime }}$) of four kinds of subdivisional works related to the prefabricated stairs are shown in Table 7.

The consumption of materials ($M_{pq}$) and machineries ($M_{pq^{{}^{\prime }}}$) per quota engineering quantity can be obtained from the above-mentioned “The Valuation Quota of Housing Construction and Decoration Engineering in Chongqing (CQJZZSDE-2018)”. In the production phase, there are two kinds of subdivisional works. Their material and machinery consumption per quota engineering are shown in Table 8.

In the transportation phase, the material and machinery consumption per quota engineering of the third kind of subdivisional works are shown in Table 9. Herein, the distance from the prefabricated stair production factory is assumed as 50 km. In the on-site construction phase, the material and machinery consumption per quota engineering of the fourth kind of subdivisional works are also shown in Table 10.

As shown in Table 10, the energy consumption ($C_{q}^{{}^{\prime }}$) per machine-team of the above machineries can be found from “Chongqing Construction Machinery Shift Quota CQJXDE-2018”.

For other subdivisional works, similar documents (Table 6, Table 7, Table 8, Table 9 and Table 10) should also be prepared based on the BIM and engineering drawing of the case. According to the carbon emission assessment model in Section 2, the carbon emission in each phase can be calculated: $E_{1-1}=E_{1-1a}+E_{1-1b}=21.656+255.580=277.236$, $E_{1-2}=0.781$, $E_{2-1}=0.011$, $E_{2-2}=7.824$, $E_{3-1}=0$, $E_{3-2}=4.382$. In turn, the total carbon emission of the case building with 22.86% prefabrication rate is E=290.504. Under the condition of other prefabrication rates, the carbon emission of the case building can also be calculated by using similar process.

## 4. Results Analysis

### 4.1. The Comparison of Total Carbon Emissions under Different Prefabrication Rates

**Figure 4:**
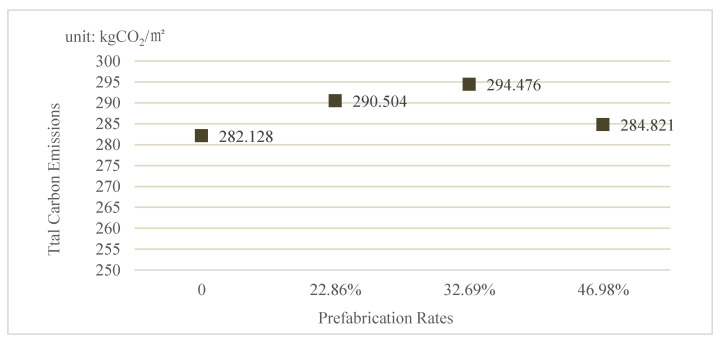
The Total Carbon Emissions of the Case Building under Different Prefabrication Rates.

The total carbon emissions of the case building under different prefabrication rates are shown in Figure 4. As can be seen, the carbon emissions per m^2^ of the case building with prefabrication rates of 0, 22.86%, 32.69%, 46.98% are 282.128 kg, 290.504 kg, 294.476 kg, and 284.821 kg, respectively. When the prefabrication rate is 0, the project is a traditional cast-in-place building.

In addition, the carbon emissions with the four prefabrication rates are different because of the different combination of components. For example, the carbon emission with rate 46.98% is less than that with rate 32.69%. The main reason is that a prefabricated partition boards is added (as shown in Table 4) in the component combination. Moreover, the prefabricated partition boards are made of concrete, while the cast-in-place partition boards are constructed by using bricks whose carbon emission factor is higher. The main materials of partition boards are changed, and in turn the carbon emission with rate 46.98% decreased than that with rate 32.69%.

### 4.2. Carbon Emission Comparison of Three Phases

**Figure 5:**
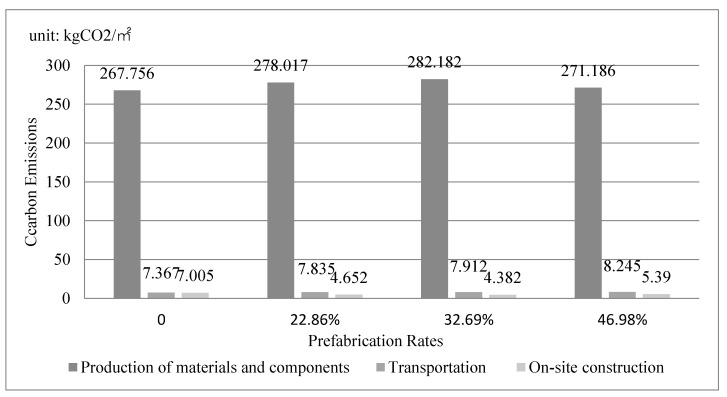
Carbon Emissions in Each Phase under Different Prefabrication Rates.

Carbon emissions in each phase under four prefabrication rates are shown in Figure 5. In the four scenarios, the production phase of components and materials generated the most carbon emissions, followed by the transportation phase and then the on-site construction phase. This is because the production phase of materials and components concentrates almost all the carbon emissions of materials in the whole construction stage, while the carbon emissions of transportation and on-site construction stage are mainly generated by machineries. Since the carbon emissions in the first phase play a dominant role in the whole construction stage, the carbon emissions from the production phase are all consistent with the total carbon emissions in four scenarios. Due to the change of material type, the carbon emission in the production phase of prefabrication rate 46.98% is significantly lower than that of rate 32.69%. With each additional prefabricated component, carbon emission in the transportation phase increases, while that in the on-site construction phase decreases.

### 4.3. Carbon Emission Comparison from the Two Sources

Table 11 shows the carbon emission ratios of two sources with the four prefabrication rates. As can be seen, the carbon emission from materials accounts for over 94% of the total carbon emission with any prefabrication rates. Because it accounts for a large proportion, the priorities of total carbon emissions and carbon emissions from materials are basically consistent.

In addition, when the prefabrication rate is 0, the carbon emission from machineries is higher than that with rate 22.86%. This is because the reinforcement of components such as stairs, beams, and slabs is connected by welding when using on-site construction, but it is changed into sleeve connection when using prefabricated components. The change in process reduces the carbon emissions from machineries.

**Figure 6:**
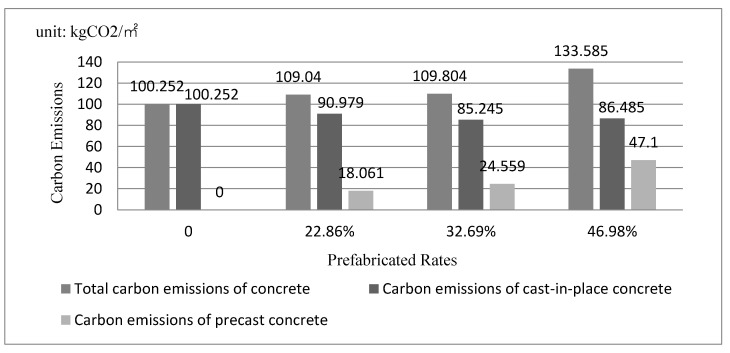
Carbon Emissions from Concrete under Different Prefabrication Rates.

In general, carbon emissions from concrete and steel take the greatest proportion in that from materials. For the case building, the carbon emissions from concrete and steel are summarized in Table 12. As can be seen, they account for over 40% under any prefabrication rates. In addition, the carbon emission from concrete and steel continue to rise with the increase in the prefabrication rate. In Figure 6, information of carbon emissions from concrete is summarized. As can be seen, the carbon emission from concrete increases with the increase in the prefabrication rate, and carbon emissions from cast-in-place concrete take the most proportion.

The carbon emissions from machineries are shown in Table 13. From the second and third rows, it can be seen that carbon emissions in the production and the transportation phase increase with the increase in the prefabrication rate. In the third phase, carbon emissions from machineries with rate of 0 are the highest because cast-in-place construction will need more machineries.

## 5. Discussions

### 5.1. Comparison with Other Studies

The utilization of prefabricated components does not reduce the carbon emission in the construction stage. However, most other existing studies [36,37,38,39,40] have drawn the opposite conclusion. This situation might be induced by the following reasons: (1) others generally compare the carbon emission of a cast-in-place building with that of another prefabricated building, and two different cases might lead to errors; however, this study pay attention on the same case; (2) as the development of prefabricated buildings in China is still in the initial stage, the materials and machineries consumption of prefabricated components is higher than that of cast-in-place components in the existing quota standard; (3) the prefabricated building is relatively new compared to that in other developed countries, and aggressive production practices may affect the carbon emissions reduction target.

In other developed countries, prefabricated buildings may have the advantages of less mistakes, less hazards, less material wastes, and higher recycling rate of components. These advantages can help reduce carbon emissions in the construction stage. However, in China, the prefabricated buildings are still in the infant stage. The related construction and management techniques are not mature enough, which makes the advantages of prefabricated buildings cannot fully work. The efficiency of reducing emissions in the construction stage by using prefabricated technology needs to be further improved in China.

### 5.2. Discussion on the Proposed Methodology

In this study, the carbon emission assessment model is developed by using the coefficient method. According to Equation (1), carbon emission activities and carbon emission factors are the main two parameters. In other related papers, carbon emission activity data are measured in real-time by using sensor data and construction records. However, in this study, the carbon emission activity data are obtained by the following process: The engineering quantities are obtained through BIM and the design drawing of the case building, and then the consumption of materials and machineries is obtained according to the quota. Therefore, the proposed method requires that the case building has design drawings with different prefabrication rates to establish BIM model and calculate the quantities.

In addition, the quota represents the average level of consumption in a specific region and will influence the results. The case building is located in Chongqing, China, and the official quota from Chongqing is used. For other cities in China or even other countries, local official quota should be accordingly utilized. Therefore, the region where a case building is located should have reasonable quota list of material and machinery consumptions. In addition, different carbon emission factors will also make the carbon emission results different. The carbon emission factors can be selected according to the average condition in the location region of the case building.

### 5.3. Suggestions on Carbon Emission Reduction

Based on the above discussion, several suggestions are put forward to use prefabricated components to reduce carbon emission in the construction stage.

#### 5.3.1. In the Production Phase

The above case has shown that the carbon emission in the production of materials and components phase take the largest proportion for the whole construction stage. Therefore, this is the most important phase to focus on.

(1) Building materials should be saved. On one hand, green energy saving building materials are more environmentally friendly, while their high cost impedes their spread and utilization. Technological innovation in related industries should be supported to produce more low priced green materials. On the other hand, the productivity of building materials can be optimized by using advanced equipment to reduce the carbon emission in the production phase.

(2) The standardization level of prefabricated components should be improved. On one hand, the utilization of prefabrication technique is relatively late in China than that of other developed countries. Although the modularization of prefabricated components can improve the efficiency of production and construction and reduce resource waste and construction costs, it is still imperfect in China, and plenty of room for improvement exists to minimize the carbon emission during the production of prefabricated components. On the other hand, mass production can improve the utilization rate of raw materials during the production process, and high standardization can provide the chance to expand production.

(3) The reuse rate of building materials should be enhanced. Compared to the traditional cast-in-place building, prefabricated building greatly saves the use of bricks, and also increases the proportion of recycled building materials. For prefabricated buildings, the recycle of steel, concrete and wood should be focused on. In addition, construction waste can be regarded as an indispensable by-product, and in turn be reused. Stakeholders in the construction industry must work together and establish a set of long-term and sustainable construction development objectives, so as to guide the reuse of building materials.

#### 5.3.2. In the Transportation Phase

(1) The transportation scheme should be optimized. The carbon emission in the transportation process is related to multiple factors, such as the distance, the load, and the path, etc. These factors should be considered together because they work together to influence the carbon emission. The factory sites of building materials and components should be overall planned at the macro level. Moreover, the path should be schemed considering real-time traffic conditions. To avoid once more transportation, the project managers should plan load volumes and time nodes in advance.

(2) The clean energy vehicle can be employed in the transportation phase. Clean energy vehicles do not consume the generally used gasoline, and become more and more environmentally friendly. They can consume less energy and in turn emit less carbons. Therefore, by improving the spread of clean energy vehicles in the transportation industry, it can provide a chance to reduce carbon emissions in the transportation phase of materials and components.

#### 5.3.3. In the On-Site Construction Phase

(1) The construction condition should be improved. In the on-site construction phase, priority should be given to the utilization of low energy consumption and reusable machineries. Compared with cast-in-place buildings, the components are more standardized, which can simplify the construction process and in turn reduce the carbon emission. However, the dispatch of machineries for prefabricated buildings is different with that for traditional cast-in-place buildings. The machinery scheduling model should be adjusted according to each specific project.

(2) Scientific management should be adopted. All construction departments should establish a site management and supervision system together to improve the awareness of low-carbon environmental protection for all workers. In terms of the reduction objective of carbon emission, specific subobjectives can be made. Such as saving building materials, reducing the loss rate of building materials, and cleaning up the construction waste in time. The introduction of BIM, Internet of Things, big data, artificial intelligence, etc., provides the possibility to support the delicacy management for the construction site, so as to help reduce carbon emissions.

## 6. Conclusions

The employment of prefabricated components is taken as a potential way to reduce carbon emissions from the construction industry. Considering the prefabrication rate may be related to the mitigation efficiency. Based on a process-based method, an assessment method was developed to compare carbon emissions of a building with multiple certain prefabrication rates in the construction stage. A dormitory building in Chongqing, China, is taken as an example to show the application of this model. The result shows that the carbon emission of prefabricated buildings is higher compared to that of traditional cast-in situ buildings. Moreover, the relation between the emissions of prefabricated buildings and the prefabrication rate is uncertain. With the improvement of the prefabrication rate, the carbon emission in the production and transportation phases gradually increase because of more prefabricated component types, while the carbon emissions of the on-site construction phase continue to decrease. From the perspective of sources, the carbon emission from building materials accounts for more than 94% of the total carbon emissions of the case building, which has the greatest impact on the total carbon emissions; the carbon emission from machineries is less than 6% and shows the increase trend with the prefabrication rate. In addition, different combination of components used in the case building induced different carbon emission reduction efficiency. Based on the research result, a detailed discussion is followed to investigate the reason why the carbon emissions do not decrease with the utilization of prefabricated units. For each phase, some improvement suggestions are made to support carbon emission reduction of buildings in the construction stage, and the production phase of materials and components should be paid most attention because of its high proportion of carbon emission.

## Figures and Tables

**Table 1:** Features of General Used Carbon Emission Assessment Methods.

Methods	Merits	Demerits	Applications
Coefficient method	The factors and sources of carbon emission is clear, and the calculation formula is simple.	When the case changes, the processing capacity is not as good as the mass balance method.	It is widely used and the conclusion is authoritative.
Mass balance method	It can distinguish the difference between each equipment and natural emission source.	The process is extremely complex.	The authority is not enough, and the accuracy of results needs to be discussed.
Actual measurement method	This method has strong pertinence and high precision.	It is too difficult to collect data.	It is rarely used.

**Table 2:** Carbon Emission Factors of Major Materials and Energies.

Carbon Sources	Carbon Emission Factors	Units	Carbon Sources	Carbon Emission Factors	Units
Electricity	1.018	kg/kw·h	Wood	83.870	kg/m^3^
Diesel	3.680	kg/kg	Iron	2.3	kg/kg
Gasoline	2.910	kg/kg	Masonry Mortar	218.14	kg/t
Water	0.414	kg/m^3^	Cement Mortar	392.65	kg/m^3^
Steel	0.367	kg/kg	Cement	0.698	kg/kg
Concrete	347.643	kg/m^3^	Standard Brick	504	kg/10^3^
Transportation	0.117	kg/km·t			

**Table 3:** Features of the Case Building.

Function	Building Category	Floors	Overall Floorage	Fire Protection Level
Dormitory	Public building	6F on ground/1F underground	15,707.68 m^2^	II

**Table 4:** The Matching of the Prefabricated rates and Components.

NO.	Prefabricated Rates	Prefabricated Components
1	0	/
2	22.86%	stairs, beams, and slabs
3	32.69%	stairs, beams, slabs, columns, and shear walls
4	46.98%	stairs, beams, slabs, columns, shear walls, and partition boards

**Table 5:** The Carbon Emission Results of the Case Building under Different Prefabrication Rates (Unit: kgCO_2_/m^2^).

Three Phases	Prefabrication Rates			
	0	22.86%	32.69%	46.98%
$E_{1-1a}$	–	21.656	29.610	53.684
$E_{1-1b}$	267.756	255.580	251.546	216.126
$E_{1-1}$	267.756	277.236	281.156	269.810
$E_{1-2}$	–	0.781	1.026	1.376
$E_{1}$	267.756	278.017	282.182	271.186
$E_{2-1}$	–	0.011	0.015	0.028
$E_{2-2}$	7.367	7.824	7.897	8.217
$E_{2}$	7.367	7.835	7.912	8.245
$E_{3-1}$	–	–	–	–
$E_{3-2}$	7.005	4.652	4.382	5.390
$E_{3}$	7.005	4.652	4.382	5.390
$E_{1}^{{}^{\prime }}$	267.756	277.248	281.171	269.838
$E_{2}^{{}^{\prime }}$	14.372	13.256	13.306	14.983
*E*	282.128	290.504	294.476	284.821

**Table 6:** Real Engineering Quantities of Subdivisional Works (Prefabrication).

Subdivisional Works			$A_{p}$	Unit	Subdivisional Works		$A_{p}$	Unit
Production	Concrete engineering	Stair	37.50	m^3^	Transportation	Stair	37.50	m^3^
		Beam	626.66	m^3^		Beam	626.66	m^3^
		Slab	264.32	m^3^		Slab	264.32	m^3^
	Steel engineering	Stair	3.46	t	On-site construction	Stair	37.50	m^3^
		Beam	83.83	t		Beam	626.66	m^3^
		Slab	21.66	t		Slab	264.32	m^3^

**Table 7:** Quota Engineering Quantities of the Prefabricated Stair.

NO.	Subdivisional Works	$A_{p}^{{}^{\prime }}$	Unit
1	Concrete engineering	10	m^3^
2	Steel engineering	1	t
3	Transportation of stairs	10	m^3^
4	Installation of stairs	10	m^3^

**Table 8:** The Material and Machinery Consumption of the First and Second Kinds of Subdivisional Works.

The First Kind of Subdivisional Works					
**Materials**	$M_{1q}$	**Unit**	**Machineries**	$M_{1q^{{}^{\prime }}}$	**Unit**
Concrete	10.100	m^3^	Portal crane (10 t)	0.230	Machine-team
Water	14.780	m^3^	Diesel dumper (1 t)	0.564	Machine-team
–	–	–	Belt conveyer (15 m*0.5 m)	0.221	Machine-team
–	–	–	Concrete mixer (350 L)	0.222	Machine-team
**The Second Kind of Subdivisional Works**					
**Materials**	$M_{2q}$	**Unit**	**Machineries**	$M_{2q^{{}^{\prime }}}$	**Unit**
Steel	1.020	t	Steel bar straightener (14 mm)	0.012	Machine-team
Water	0.290	m^3^	Steel bar cutter (40 mm)	0.075	Machine-team
–	–	–	Steel bar bender (40 mm)	0.150	Machine-team
–	–	–	Tributary arc welder (32 kV·A)	0.373	Machine-team
–	–	–	Butt welder (75 kV·A)	0.068	Machine-team
–	–	–	Electric welding machine (75 kV·A)	0.069	Machine-team
–	–	–	Welding rod drying box (450∗350∗450)	0.042	Machine-team

**Table 9:** The Material and Machinery Consumption of the Third and Fourth Kinds of Subdivisional Works.

The Third Kind of Subdivisional Works					
**Materials**	$M_{3q}$	**Unit**	**Machineries**	$M_{3q^{{}^{\prime }}}$	**Unit**
Wood	0.010	m^3^	Auto crane (5 t)	0.522	Machine-team
Steel wire rope	0.320	kg	Motor truck (8 t)	3.813	Machine-team
**The Fourth Kind of Subdivisional Works**					
**Materials**	$M_{4q}$	**Unit**	**Machineries**	$M_{4q^{{}^{\prime }}}$	**Unit**
Concrete	0.160	m^3^	Crawler crane (15 t)	0.073	Machine-team
Cement Mortar	0.120	m^3^	Wheel crane (20 t)	0.022	Machine-team
Wood	0.015	m^3^	Concrete mixer (350 L)	0.018	Machine-team
Iron	13.610	kg	Mortar mixer (200 L)	0.018	Machine-team
Water	4.420	m^3^	Tributary arc welder (32 kV·A)	1.362	Machine-team

**Table 10:** The Energy Consumption per Machine-team of Above Machineries.

Machineries	Energies	$C_{q}^{{}^{\prime }}$	Unit	Machineries	Energies	$C_{q}^{{}^{\prime }}$	Unit
Portal crane (10 t)	Electricity	88.29	kW·h	Butt welder (75 kV·A)	Electricity	122.00	kW·h
Diesel dumper (1 t)	Diesel	6.03	kg	Electric welding machine (75 kV·A)	Electricity	154.63	kW·h
Belt conveyer (15 m∗0.5 m)	Electricity	20.58	kW·h	Welding rod drying box (450∗350∗450)	Electricity	6.70	kW·h
Concrete mixer (350 L)	Electricity	43.52	kW·h	Auto crane (5 t)	Gasoline	23.30	kg
Steel bar straightener (14 mm)	Electricity	11.90	kW·h	Motor truck (8 t)	Diesel	35.49	kg
Steel bar cutter (40 mm)	Electricity	32.10	kW·h	Crawler crane (15 t)	Diesel	29.52	kg
Steel bar bender (40 mm)	Electricity	12.80	kW·h	Wheel crane (20 t)	Diesel	41.51	kg
Tributary arc welder (32 kV·A)	Electricity	96.52	kW·h	Mortar mixer (200 L)	Electricity	8.61	kW·h

**Table 11:** Carbon Emission Ratios under Different Prefabrication Rates.

Ratios	Prefabrication Rates			
	0	22.86%	32.69%	46.98%
$E_{1}^{{}^{\prime }}/E$	94.9%	95.4%	95.5%	94.7%
$E_{2}^{{}^{\prime }}/E$	5.1%	4.6%	4.5%	5.3%

**Table 12:** Carbon Emissions of Key Materials under Different Prefabrication Rates.

Carbon Emissions	Prefabrication Rates				Unit
	0	22.86%	32.69%	46.98%	
From all materials	267.756	277.248	282.171	269.838	kgCO_2_/m^2^
From concrete and steel (key materials)	116.007	124.617	126.081	150.965	kgCO_2_/m^2^
The ratio of key materials and all materials	43.33%	44.95%	44.68%	55.95%	/

**Table 13:** Carbon Emissions from Machineries under Different Prefabrication Rates (Unit: kgCO_2_/m^2^).

Carbon Emissions	Prefabrication Rates			
	0	22.86%	32.69%	46.98%
$E_{2}^{{}^{\prime }}$	14.372	13.256	13.306	14.983
$E_{1-2}$	——	0.781	1.026	1.376
$E_{2-2}$	7.367	7.824	7.897	8.217
$E_{3-2}$	7.005	4.652	4.382	5.390
